# Engineering Biomaterials and Approaches for Mechanical Stretching of Cells in Three Dimensions

**DOI:** 10.3389/fbioe.2020.589590

**Published:** 2020-10-14

**Authors:** Weiwei Zhang, Guoyou Huang, Feng Xu

**Affiliations:** ^1^Faculty of Materials Science and Chemistry, China University of Geosciences, Wuhan, China; ^2^Key Laboratory of Biorheological Science and Technology (Chongqing University), Ministry of Education, Chongqing University, Chongqing, China; ^3^Department of Engineering Mechanics, School of Civil Engineering, Wuhan University, Wuhan, China; ^4^Bioinspired Engineering and Biomechanics Center, Xi’an Jiaotong University, Xi’an, China; ^5^The Key Laboratory of Biomedical Information Engineering of Ministry of Education, School of Life Sciences and Technology, Xi’an Jiaotong University, Xi’an, China

**Keywords:** mechanobiology, stretch, tissue engineering, hydrogels, cell mechanotransduction

## Abstract

Mechanical stretch is widely experienced by cells of different tissues in the human body and plays critical roles in regulating their behaviors. Numerous studies have been devoted to investigating the responses of cells to mechanical stretch, providing us with fruitful findings. However, these findings have been mostly observed from two-dimensional studies and increasing evidence suggests that cells in three dimensions may behave more closely to their *in vivo* behaviors. While significant efforts and progresses have been made in the engineering of biomaterials and approaches for mechanical stretching of cells in three dimensions, much work remains to be done. Here, we briefly review the state-of-the-art researches in this area, with focus on discussing biomaterial considerations and stretching approaches. We envision that with the development of advanced biomaterials, actuators and microengineering technologies, more versatile and predictive three-dimensional cell stretching models would be available soon for extensive applications in such fields as mechanobiology, tissue engineering, and drug screening.

## Introduction

**FIGURE 1:**
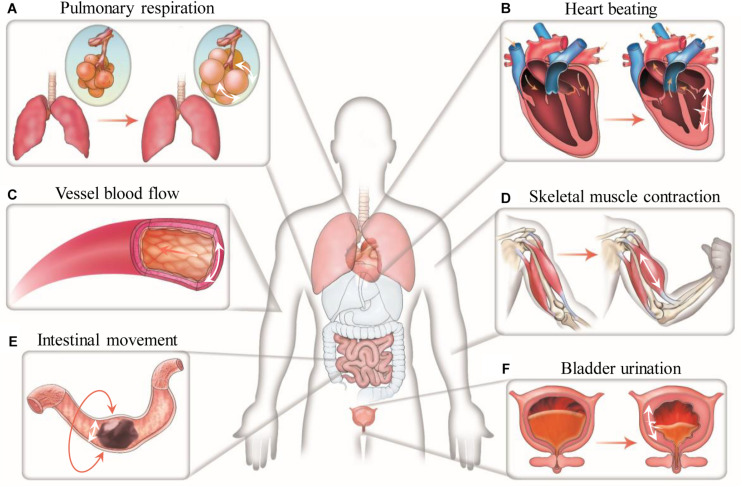
Mechanical stretch in the human body. Representative stretching forces in different human tissues and organs are indicated by white arrows. **(A)** Cells in the alveoli undergo cyclic dilatational stretching during pulmonary respiration. **(B)** Cells in the myocardium experience cyclic circumferential and longitudinal stretching during heart beating. **(C)** Cells in the vessel wall are continuously subjected to circumferential stretching due to the action of blood pressure. **(D)** Cells in the skeletal muscle experience uniaxial stretching when moving the body. **(E)** Cells in the intestinal wall undergo circumferential stretching during intestinal peristalsis. **(F)** Cells in the bladder wall experience circumferential and longitudinal stretching at the time of urination.

Cells in the human body experience various mechanical forces such as tensile, shear, compressive, torsional and hydrostatic forces, with mechanical features depending on specific tissue types, development stages and body conditions (Polacheck et al., 2013; Giulitti et al., 2016; Huang G. et al., 2019). Specially, cells in the lung and heart are cyclically subjected to mechanical stretch during breathing and heart beating (Figure 1). Such stretching force plays important roles in regulating the behaviors of lung and heart cells, and thus the development and performances of the lung and heart (Sheehy et al., 2012; Liu Z. et al., 2016; Stoppel et al., 2016; Watson et al., 2019). Mechanical stretch can be also commonly found in many other tissues or organs such as skeletal and smooth muscles, tendon, vessel, intestine, bladder and cartilage, etc., prominently regulating the behaviors of cells in these systems (Qi et al., 2016; Landau et al., 2018; Rinoldi et al., 2019). For instance, mechanical stretch has been widely demonstrated to promote the maturation and growth of muscles (Li et al., 2015; Weinberger et al., 2017). Intestinal stretch as induced by food-intake was recently found to be able to stimulate cells in the intestinal wall to generate satiety signals for feeding regulation (Bai et al., 2019).

Mechanical stretch can be originally generated from external loading or internal active contraction, and may specifically elicit cell responses different from that induced by other mechanical stimuli (Maul et al., 2011; Zhong Z. et al., 2011). Almost all aspects of cell behaviors, including cell shape, orientation, proliferation, secretion, gene and protein expression, lineage differentiation and apoptosis, have been found to be regulated by mechanical stretching, with actual effects depending on cell types, stretch parameters, and culture conditions (Li Y. et al., 2014; Xu et al., 2016; Chen et al., 2018; He et al., 2018). By responding and adapting to mechanical stretching, cells can maintain their mechanical integrity and modulate their tensional state to sustain mechanical equilibrium, i.e., tensional homeostasis (Brown et al., 1998; Humphrey et al., 2014; Cheng et al., 2017). The disruption of tensional homeostasis usually leads to mechanical force-associated diseases, including defective morphogenesis or pathological dysfunctions such as fibrosis and cancer (Cambré et al., 2018; Bonnevie et al., 2019; Boudou et al., 2019). For example, chronically elevated cyclic stretch can induce abnormal proliferation and migration of vascular smooth muscle cells to mediate pathological vascular remodeling during hypertension (Qi et al., 2010). As a recent excellent example, Sainz de Aja and Kim (2020) and Wu et al. (2020) found that in idiopathic pulmonary fibrosis (IPF, the most common type of lung fibrosis), loss of Cdc42 function in alveolar stem cells (AT2 cells) results in impaired alveolar regeneration and consequently exposes AT2 cells to sustained elevated mechanical tension. Such aberrant elevated and likely spatial-specifically distributed mechanical tension generates an activation loop of TGF-ß signaling in AT2 cells in a spatially regulated manner, driving periphery-to-center progression of IPF.

Various biomaterials and approaches have been developed for mechanical stretching of cells, most of which have been performed on two-dimensional (2D) substrates (Kurpinski et al., 2006; Yung et al., 2009; Cui et al., 2015; Wang et al., 2015; Kamble et al., 2016). In such studies, monolayer of cells is usually cultured on the surface of elastic membranes made of elastomer [typically polydimethylsiloxane (PDMS)] or hydrogels. By inducing expanding or bending deformation of the elastic membranes, mechanical stretch can be generated and applied to the cells cultured on them (Huh et al., 2010; Faust et al., 2011; Mann et al., 2012; Jiang et al., 2018). Various approaches, commonly including motor-driven, indentation, pneumatic actuation, magnetic and electromagnetic actuation, have been developed to induce mechanical deformation of the elastic membranes. Thanks to the simple configuration and easy manipulation of elastic membranes, the responses of cells to mechanical stretch of diverse parameters varying in stretch mode (uniaxial, biaxial, and equiaxial), stretch waveform (static, sinusoidal, and ramp), insertion of rest periods, strain magnitude, rate or frequency, have been systematically investigated (Balestrini and Billiar, 2009; Zhong Y. et al., 2011; Zhao G. et al., 2019). In addition, specific dielectric (Poulin et al., 2016, 2018; Imboden et al., 2019) and electrochemical (Svennersten et al., 2011; Guan et al., 2018) actuators have been developed to achieve ultra-fast dynamic cell stretching or high-throughput single cell stretching, respectively. Moreover, local stretch of subcellular or even molecular structures has been reported by employing specially designed magnetic tweezers (Tajik et al., 2016; Sun et al., 2020), optical tweezers (Han et al., 2018, 2020) or other micromanipulators (Kim et al., 2009; Scheiwe et al., 2015). These studies have dramatically benefited the understanding of mechanical stretch-associated mechanobiology and cell mechanotransduction.

**FIGURE 2:**
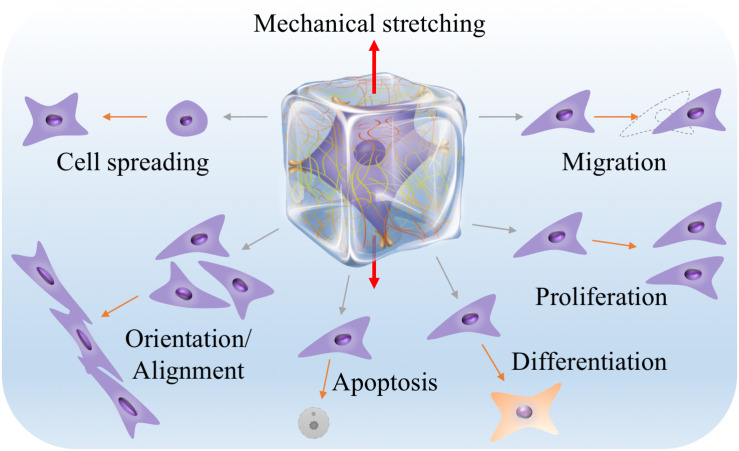
Many aspects of cell behaviors, including cell spreading, migration, orientation or alignment, proliferation, apoptosis and lineage differentiation, can be influenced by 3D mechanical stretching. The middle illustration was reprinted with permission from (Huang et al., 2017).

Nevertheless, increasing evidence shows that the dimensionality of the microenvironment where cells reside in may greatly influence the responses of cells to mechanical stimuli including stretch stimulation (Riehl et al., 2012; Caliari et al., 2016). Since majority of cells in the human body are embedded in complex three-dimensional (3D) extracellular matrix (ECM), it is reasonable to expect that, as have been already widely demonstrated, studying cell behaviors in a 3D engineered microenvironment can better recapture native cell responses than in two dimensions. Similar to but could be different from the findings from 2D studies, stretch forces in 3D have been found to influence many aspects of cell behaviors, including cell spreading, migration, orientation or alignment, proliferation, apoptosis and lineage differentiation (Figure 2). Therefore, engineering biomaterials and approaches for mechanical stretching of cells in three dimensions have attracted increasing interests, particularly in the engineering of 3D tissue constructs for applications in basic research, tissue engineering, regenerative medicine and drug screening (Zimmermann et al., 2006; Eyckmans and Chen, 2017; Huang et al., 2017). To allow 3D cell culture, cells are usually encapsulated in hydrogels or 3D scaffolds. Stretch forces are thus transmitted to the encapsulated cells through deformed meshes of hydrogels. Unlike stretching of cells on 2D elastic membranes, stretching of cells in 3D hydrogels may encounter some practical issues such as difficult to anchor and stretch hydrogels, limitation in controlling stretch parameters, insufficient supply of nutrients and oxygen, and challenges in 3D mechanical and biological characterizations (Riehl et al., 2012). Despite all these, significant efforts and progresses have been made to mechanically stretch cells and study their responses in a 3D engineered microenvironment over the past decade.

In this article, we briefly review the state-of-the-art advances in the engineering of biomaterials and approaches for mechanical stretching of cells in three dimensions. We first discuss biomaterial considerations for mechanical stretching of cells in three dimensions from mechanical, structural and biochemical aspects, respectively. We then summarize research status of hydrogel anchoring and actuation approaches for mechanical stretching of cells in three dimensions. Finally, concluding remarks and perspectives are given.

## Engineering Biomaterials for Mechanical Stretching of Cells in Three Dimensions

**FIGURE 3:**
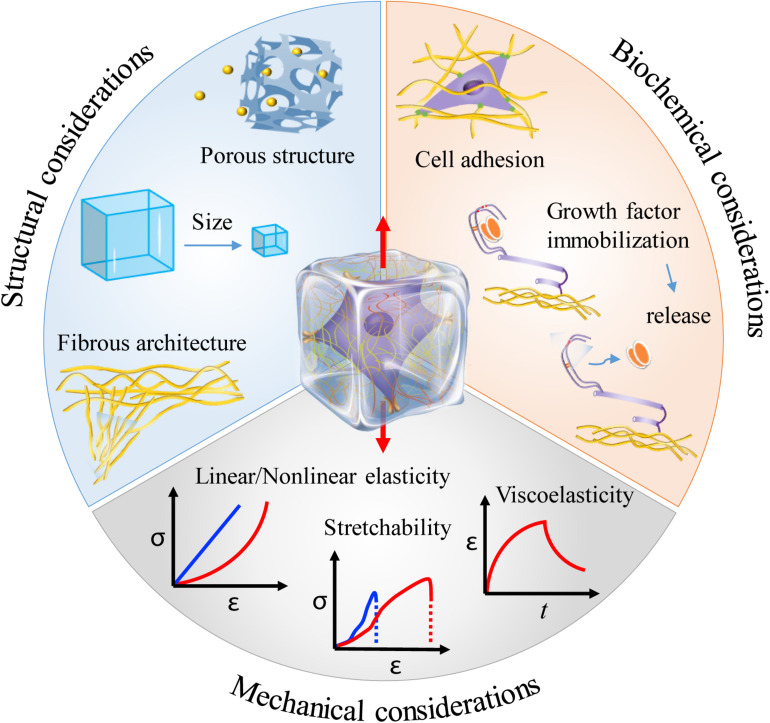
Material considerations when engineering hydrogels for mechanical stretching of cells in three dimensions. Hydrogels can provide diverse mechanical, structural and biochemical cues that may greatly affect cell responses to mechanical stretching in three dimensions. The middle illustration was reprinted with permission from Huang et al. (2017).

Most of the cells *in vivo*, if not all, inherently adhere to the ECM or cells for survival and growth. The ECM provides not only structural support but also various mechanical and biochemical cues for directing cell behaviors. This has recently been comprehensively reviewed in the literature (Huang et al., 2017). To mimic the ECM, hydrogels have been widely used in 3D cell culture. From the aspects of mechanical stretching, hydrogels can not only transmit stretch forces to the resident cells, forcing cells to stretch, but also respond to mechanical stretch itself via structural remodeling and biochemical molecule regulation, all of which can have profound effects on cell behaviors (Vader et al., 2009; Gaul et al., 2018; Liu et al., 2020; Pei et al., 2020). It is therefore reasonable to include mechanical, structural and biochemical considerations when engineering hydrogels for mechanical stretching of cells in three dimensions (Figure 3; Li et al., 2017; Davidson et al., 2020).

### Mechanical Considerations

Mechanical properties of the hydrogels used in 3D cell culture determine the stress (the force over a given area) or strain (a description of deformation in terms of relative displacement of particles in the body) that can be generated in hydrogels under defined loading forces. The most studied and the primary considered mechanical property of the hydrogels is stiffness – the extent to which an object resists deformation in response to an applied force (Engler et al., 2006; Li et al., 2017; Wang M. et al., 2019). In practice, stiffness is usually characterized by elastic modulus or Young’s modulus (the slope of the stress-strain curve in the linear elastic region). High stiffness may shield cells from high strain by resisting deformation. However, it should be noted that stiffness itself is widely found to play critical roles in regulating cell behaviors. For example, as the most widely distributed cells in the human body, fibroblasts orchestrate ECM homeostasis by responding to diverse microenvironment cues including matrix stiffness (van Putten et al., 2015). In the heart, pathological development of cardiac fibrosis typically leads to increased matrix stiffness and thus reduced local mechanical strains under heart beating, which cooperatively regulate fibroblast activation and fibrosis development (Yong et al., 2015). Interestingly, a recent work reported that cell mechanosensing is not only regulated by substrate stiffness, but also by strain energy (Panzetta et al., 2019). This further complicates the interpretation of cell mechanosensing and the design of biomaterials for engineering the cell mechanical microenvironment, since straining biomaterials usually leads to corresponding changes in matrix stiffness, showing a non-linear strain–stress relationship as will be discussed below. In this case, it will be difficult to decouple the effects of strain from stiffness. Fortunately, advanced hydrogels with a linear strain–stress relationship in a wide strain range are available (Li et al., 2016a,b).

When applied for mechanically stretching cells in three dimensions, the stretchability of hydrogels should be taken into account, especially when large strains are expected such as for investigating tendons and muscles. Possibly the simplest ways of enhancing hydrogel stretchability are by reducing polymer concentration or by increasing characteristic network length. However, they are usually accompanied by a significant decrease of hydrogel stiffness, which is not expected in most cases. Therefore, certain efforts that can be generally classified into material composition- and structure-based approaches have been made to develop highly stretchable and/or tough (the ability to resist fracture by absorbing and dissipating mechanical energy) hydrogels (Li J. et al., 2014; Feng et al., 2016). In the material composition-based approach, components with complementary properties are combined to form hybrid hydrogels, in which one component endows the hydrogels high stretchability and the other component mainly works to absorb or dissipate mechanical energy (Sun et al., 2012). While the fabrication of such hybrid hydrogels often require toxic chemicals and/or harsh processing conditions, recent studies did report some biocompatible hybrid hydrogels [e.g., poly(ethylene glycol)/sodium alginate hydrogels (Hong et al., 2015)] that can be used for cell encapsulation and long-term 3D cell culture, maintaining high stretchability and toughness. In the structure-based approach, hydrogels are fabricated into special structural forms that can bear large deformations. For example, when fabricated into microscales, hydrogels of gelatin methacrylate (GelMA) can be stretched up to three times of its initial length (Li et al., 2016b). Further improvement can be achieved by fabricating hydrogels into hierarchical helical structures (Li et al., 2019).

As mentioned above, one of the important mechanical features of many native ECM and natural biomaterials (e.g., type I collagen and fibrin) is the non-linear stress-strain relationship, mostly exhibiting strain-stiffening behavior (i.e., the stiffness is not constant but increases with increasing strain) when subjected to large deformations (Storm et al., 2005; Licup et al., 2015). As for type I collagen and fibrin, the non-linear strain-stiffening behavior was found to be dependent on strain history (Münster et al., 2013). The fibrous nature of these ECM proteins, including recruitment and alignment, is central to their effects. Moreover, the integration of other material components (e.g., polysaccharide matrix) with these fibrous proteins may have profound effects on their non-linear strain-stiffening behavior (Burla et al., 2019). Cells can actively use stiffening to strain their microenvironment and generate positive mechanical feedback from the ECM to modulate their functions (Jaspers et al., 2014; Das et al., 2016; Hall et al., 2016; Han et al., 2018). From mechanical stretching aspects, hydrogels with non-linear strain-stiffening property enables long-range transmission of mechanical forces and thus long-range cell-cell communications (Wang H. et al., 2014; Sopher et al., 2018). Moreover, such property is believed to be important in preventing overlarge tissue deformation, maintaining tissue integrity and thus shielding cells from overstretching (Wen and Janmey, 2013). Nevertheless, cells of diverse types, including fibroblasts and epithelial cells, may still subject to severe deformations even at physiological conditions as they engage in migration, invasion and lumen dilation (Trepat et al., 2007). Interestingly, these cells are not just passively receiving mechanical forces from external loading. They can respond to mechanical forces by dynamically adapting their cytoskeleton networks to withstand substantial deformations (Fletcher and Mullins, 2010). For instance, actin filaments and microtubules can yield and disassemble under moderate strains, while vimentin containing intermediate filaments forms a stretchable, hyperelastic network that can maintain cell viability at large deformations and increase the stretchability, strength and toughness of the cytoplasm (Hu et al., 2019). Therefore, epithelial cells can undergo extreme stretching and enter a state of non-linear superelasticity (the deformation increases without a corresponding increase in tension) (Latorre et al., 2018).

Many hydrogels exhibit not only solid- (elastic) but also fluid-like (viscous) characteristics when undergoing deformation (Pryse et al., 2003; Babaei et al., 2016; Nam et al., 2016a). The viscosity of a hydrogel may arise from weak bond dissolution, molecular slipping, polymer disentanglement or protein unfolding, etc. Such viscoelastic property endows hydrogels the ability to dissipate energy, allowing stress relaxation (a time-dependent decrease in stress under a constant strain) or creep (a time-dependent increase in deformation under a constant stress). Under dynamic loading conditions, the viscoelasticity of hydrogels can be significantly depending on strain rate. Remarkable efforts and progress have been made in recent years toward the development of advanced viscoelastic hydrogels, with certain results showing that the hydrogel viscoelasticity may greatly influence cell morphology, proliferation and differentiation (Chaudhuri et al., 2015, 2016; Bauer et al., 2017; Charrier et al., 2018; Gong et al., 2018; Lou et al., 2018; Huang D. et al., 2019; Cantini et al., 2020). In addition to viscoelasticity, some hydrogels (especially reconstituted fibrous hydrogels such as type I collagen) may undergo plastic deformation (a non-reversible permanent change in shape) when subjected to mechanical loadings (Kim et al., 2017; Ban et al., 2018; Ming et al., 2020). Such property can be explored by contractile cells to remodel the ECM, inducing local persistent alignment and densification of ECM fibers for facilitating cell migration and fibrosis development (Nam et al., 2016b; Wisdom et al., 2018).

### Structural Considerations

In 3D cell culture, a great challenge is to maintain high cell viability. Bulk hydrogels used for encapsulating cells usually have a diffusion limit of several hundred micrometers, beyond which the cells may suffer from significant exhaustion of oxygen and nutrients (Huang et al., 2011). Dynamic mechanical stretching has been found to be able to facilitate oxygen and nutrient transport in hydrogels, however, usually it could not be sufficient to guarantee cell survival (Vaughan et al., 2013). Therefore, it is necessary to introduce additional macroscale porous or even microfluidic structures into bulk hydrogels to enable convective mass transport (Choi et al., 2007; Huang et al., 2013). However, attention should be paid to avoid structural collapses, maintaining hydrogel mechanical stability under both perfusion culture and mechanical stretching (Huang et al., 2012). Perhaps the most effective way to overcome diffusion limitation is to reduce the size of cell-laden hydrogels. As a result, various microengineering technologies have been developed to fabricate microscale engineered tissues (i.e., microtissues), which not only overcome diffusion limitation, but also bring benefits such as the save of materials and cells, high throughput, as well as enhanced mechanical stretchability as already mentioned in section “Mechanical Considerations” (Wang L. et al., 2014; Guven et al., 2015; Zhu et al., 2017). In practice, it may cause more difficulties in mechanically stretching microtissues. Nevertheless, with the development of advanced microfabrication and actuation technologies, various approaches have been explored for simultaneously stretching multiple microtissues (more discussions can be found in section “Approaches for Stretching Three-Dimensional Engineered Tissue Constructs”).

A key feature of the native ECM is the fibrous structure that is mainly determined by fibrous collagen, fibrin, fibronectin and elastin networks. Such fibrous structures have been found to dramatically contribute to control ECM mechanical properties such as strain-stiffening and viscoelasticity (Prince and Kumacheva, 2019; van Oosten et al., 2019). Cells can sense fiber features (e.g., diameter, length, density and direction), actively remodel the fibrous networks via cell contraction, and respond by adjusting their contractility, migration, alignment and growth (Holmes et al., 2018; Brauer et al., 2019; Wang W. Y. et al., 2019). Moreover, recent studies have identified a particular important role of the fibrous networks in long-distance cell-cell communications and collective behaviors (Han et al., 2018; Sarker et al., 2019; Liu et al., 2020). While some reconstituted protein-based biopolymers (e.g., type I collagen) can spontaneously self-assemble into fibrous structures under mild conditions, many widely used biopolymers [e.g., gelatin, sodium alginate, hyaluronic acid (HA)] and most synthetic hydrogels often lack fibrous structural features. Therefore, various approaches, e.g., self-assembly, phase separation and electrospinning, have been developed to generate synthetic fibrous constructs to mimic the architecture of fibrous ECMs (Zhao et al., 2015; Prince and Kumacheva, 2019). An excellent example is the electrospun HA-based fibrous hydrogels, which have been applied to study the roles of local fiber recruitment and fiber mechanics on cell spreading, proliferation, focal adhesion signaling and myofibroblast differentiation (Kim et al., 2013; Baker et al., 2015; Cao et al., 2017; Davidson et al., 2019). Interestingly, mechanical stretch cannot only direct the organization of single fibrous network but also regulate the interactions between both cell-matrix and different fibrous components (Vader et al., 2009; Kubow et al., 2015). Particularly, mechanical stretch has been found to modulate enzymatic degradation of collagen by sequestrating cleavage sites on collagen fibers (Bhole et al., 2009; Ghazanfari et al., 2016; Gaul et al., 2018). These further emphasize the importance of using fibrous hydrogels when engineering the stretching microenvironment of cells.

### Biochemical Considerations

Biochemical cues have been extensively investigated in cell biology and tissue engineering. Mechanical stimuli can cooperatively or oppositely work with biochemical cues to orchestrate cell behavior (Salameh et al., 2010; Wan et al., 2019). From the perspective of biomaterial design, hydrogels may provide some specific biochemical properties that can influence the effect of mechanical stretch on cells. Specifically, hydrogels with different cell adhesive properties (usually determined by the presence of chemical functional groups or cell adhesion ligands on hydrogel networks) can show different capacities in transmitting mechanical forces or strains to cells encapsulated in them. Generally, the higher affinity between hydrogels and cells, the more efficiency of transmitting mechanical forces or strains by the hydrogels. This could be a dominant role of ECM sub-types and different biochemically modified hydrogels in regulating the responses of cells to mechanical stretch (Atance et al., 2004; Watson et al., 2014).

On the other hand, hydrogels can be engineered to sequester or immobilize growth factors by mimicking the performance of some ECM macromolecules, such as glycosaminoglycans (GAGs) (Gama et al., 2006; Lienemann et al., 2012; Hettiaratchi et al., 2016). These growth factors can be released and activated in response to mechanical stretching of hydrogels induced by cell contractile forces or external mechanical loadings, thus playing important roles in regulating cell mechanobiological responses. As a typical example, transforming growth factor-β1 (TGF-β1, a polypeptide cytokine belonging to the TGF superfamily that performs many functions in many cell types) is generally secreted and stored in the matrix as part of a large latent complex bound to the latent TGF-β binding protein (LTBP). When the applied mechanical stretching is above a certain threshold, TGF-β1 can be liberated and activated, establishing a mechanical checkpoint and contributing to many biological processes such as the progression of tissue repair and fibrosis (Maeda et al., 2011; Hinz, 2015; Froese et al., 2016; Walker et al., 2020).

## Approaches for Stretching Three-Dimensional Engineered Tissue Constructs

**FIGURE 4:**
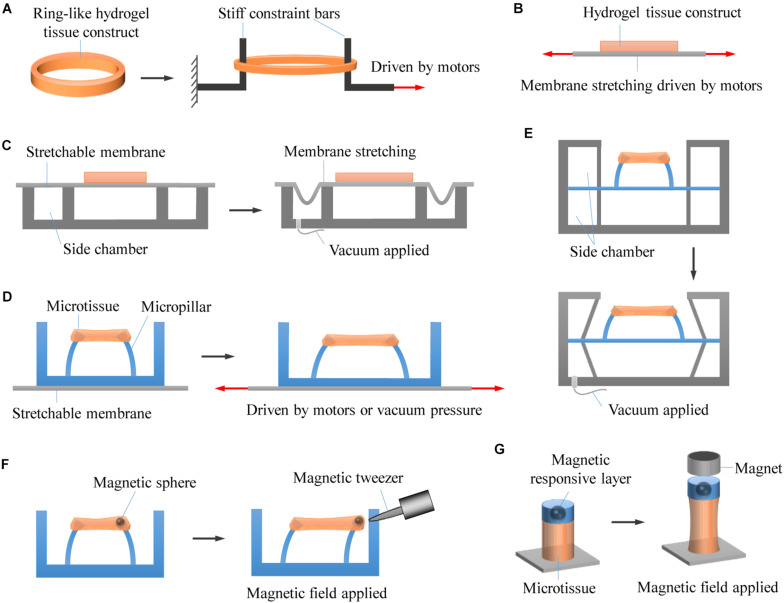
Representative approaches for stretching 3D engineered tissue constructs. **(A)** Hydrogel tissue constructs are fabricated into macroscale ring-like shapes, physically anchored with two stiff rods, and actuated to undergo stretching by using motor-driven approach. **(B)** Hydrogel tissue constructs are chemically bonded on stretchable membranes that are driven to stretch by using step motors. **(C)** Hydrogel tissue constructs are chemically bonded on stretchable membranes that are driven to stretch by applying vacuum pressure. **(D)** Hydrogel microtissue constructs are constrained by elastic micropillars. The distance between the micropillars changes when the underlying elastomeric membranes are stretched by either motor-driven or pneumatic actuation approaches, leading to deformation of the constrained microtissues. **(E)** Pneumatic actuation of micropillar-anchored microtissues by applying vacuum pressure to the side chambers of microtissues. **(F)** The microtissues are magnetically actuated by applying a non-uniform magnetic field to attract the magnetic microspheres fixed to the micropillars. **(G)** Enabling magnetic actuation by fabricating magnetic responsive layer onto photopatterned microtissues that chemically anchored on glass substrates.

While widely used for engineering 3D tissue constructs, it is still challenging to stretch cell-encapsulating hydrogels in a controllable way. Nevertheless, significant efforts have been made to anchor and actuate hydrogels for mechanically stretching hydrogel-based 3D engineered tissue constructs (Figure 4).

### Anchoring Approaches

To enable mechanical stretching, engineered tissue constructs need to be anchored first to prevent unexpected slipping movement. Since 3D engineered tissue constructs are usually constructed by encapsulating cells into hydrogels, it is a big challenge to anchor such hydrogels. Unlike elastomers and rubbers that can be easily clamped for mechanical stretching tests, hydrogels used for engineering 3D tissue constructs are usually difficult to be anchored with conventional clamps. This is mainly due to their highly hydrated and relatively soft properties that make them susceptible to slippage, deformation or breakage under clamping and stretching (Tomei et al., 2009; Galie and Stegemann, 2011). To robustly anchor hydrogels, various approaches have been explored that can be generally classified into chemical anchoring and physical anchoring.

In chemical anchoring, hydrogels are usually covalently bonded to modified or activated anchoring surfaces during crosslinking. For example, GelMA provides reactive methacrylic groups that can be photo-crosslinked to 3-(trimethoxysilyl)propyl methacrylate-modified glass substrates when exposed to ultraviolet light in the presence of photoinitiators (Van den Bulcke et al., 2000; Nichol et al., 2010; Hsieh et al., 2014). However, such approach is only applicable to some specific types of hydrogels. Similar approach has been applied for achieving tough bonding of hydrogels to amino-silane functionalized substrates of diverse solids by using EDC-Sulfo NHS chemistry (Yuk et al., 2016). However, it could not be used in the presence of cells due to the involvement of toxic chemicals and/or harsh conditions. While certain adhesives can chemically bond hydrogels to anchoring surfaces, this strategy involves the issues of weak bonding, low biocompatibility and/or slow adhesion formation (Pang et al., 2010; Mihic et al., 2014). Recently, Yuk et al. (2019) developed an interesting dry double-sided tape (DST) for rapid and strong adhesion of wet tissues and devices. Such DST could be employed for anchoring hydrogels in the presence of cells.

In contrast to chemical anchoring, physical anchoring does not apply chemical crosslinking but uses physical constraint to anchor hydrogels. For macroscale tissue constructs, this can be achieved by special designs (e.g., card slot and/or porous designs) of the anchors (Roeder et al., 2002; Gould et al., 2012). In the card slot design, the ends of the hydrogels are fabricated into designed shapes that can be physically locked by card slot anchors. This method, however, can be only used for stiff enough hydrogels. In the porous design, hydrogel precursor solution is firstly allowed to infiltrate into porous anchors and then crosslinked to form hydrogels with the ends integrated with the porous anchors. Both card slot and porous designs could involve troublesome operations and are not suitable for small-scale hydrogel tissue constructs. In addition, hydrogel tissue constructs have also been fabricated into ring- or runway-like shapes, which can then be simply hitched by two rods or pins for further mechanical stretching (Wakatsuki et al., 2000; Zimmermann et al., 2000, 2002; Berry et al., 2003; Asnes et al., 2006; Lee et al., 2012; Rinoldi et al., 2019). Inspired by this, and through tactfully exploiting the phenomenon of cell-induced collagen remodeling, Legant et al. (2009) developed a micropillar-based geometrical confinement approach that enables high-throughput fabrication of constrained microscale tissue constructs (i.e., engineered microtissues) of cell-encapsulating collagens. The custom-designed elastic micropillars (termed as microfabricated tissue gauges) are not only used to constrain the engineered microtissues but also to report forces generated by them in real time. Such approach has attracted great interests in the past decade (Jason et al., 2014; Liu et al., 2014; Mills et al., 2017; Ronaldson-Bouchard et al., 2018; Chen and Zhao, 2019). Recently, a similar approach has been reported by using elastic microwires instead of micropillars to constrain and monitor microtissues, showing great promises in heteropolar microtissue construction and disease modeling (Mastikhina et al., 2019; Wang E. Y. et al., 2019; Zhao Y. et al., 2019).

### Actuation Approaches

When anchored, hydrogels can be actuated to undergo deformation, generating stretch stimulus to the encapsulated cells. While various actuation approaches have been developed to stretch single or monolayer cells, certain of them have not been adopted for stretching hydrogel-based 3D tissue constructs, mainly due to their limited driving force or poor strain controllability. Currently, the main approaches for driving hydrogel deformation can be generally classified into three types: motor-driven approach, pneumatic actuation, and magnetic actuation (Table 1).

**TABLE 1:** A brief comparison and summary of the main actuation approaches.

**Approaches**	**Comments**
**Motor-driven approach**	**Advantages:** particularly suitable for uniaxial stretching and for engineering large tissue constructs, allow multiple loading modes and precise control over strain amplitude and strain rate
	**Disadvantages:** potential contamination, difficult to simultaneously meet miniaturization, compartmentalization and high-throughput requirements

**Pneumatic actuation**	**Advantages:** simple setup, readily to incorporate with microfluidic technologies
	**Disadvantages:** limited in actuation rate, could suffer from non-uniform strain distribution

**Magnetic actuation**	**Advantages:** enable actuation in a non-contact and remote way, with the ability to measure microtissue stiffness and to change boundary stiffness in real time
	**Disadvantages:** could be cumbersome to design and integrate the magnetic actuation system, potential unwanted magnetic effect on cells

Early studies that stretch macroscale tissue constructs usually use custom-designed motor-driven systems. Specifically, hydrogels encapsulating cells are anchored by clamps or stiff bars, which are connected to driving motors. The movement of driving motors thus leads to the deformation of cell-laden hydrogels (Fink et al., 2000; Wakatsuki et al., 2000; Powell et al., 2002). This approach is particularly suitable for uniaxial stretching. Biaxial stretching can also be achieved by perpendicularly equipping two driving motors, providing that the hydrogel tissue constructs are appropriately anchored. Benefit from the advantages of driving motors, such approach allows multiple loading modes, and precise control over strain amplitude and strain rate. Moreover, it is readily to incorporate mechanical transducers for measuring tensile forces. However, the physical contact between the driving system and the cell culture medium may involve the potential of contamination. In addition, it is suitable for engineering large tissue constructs but difficult to meet miniaturization and high-throughput requirements for drug screening applications.

Compared to directly stretching hydrogels, stretching elastomeric membranes is easier to implement and may avoid direct contact with cell culture medium. When chemically anchored on an elastomeric membrane, hydrogel tissue constructs can undergo deformation with the membrane, which can be achieved by using either motor-driven or pneumatic actuation approaches (Riehl et al., 2012; Doryab et al., 2019). Due to the substantial advantage of simple setup, pneumatic actuation has been commonly used (as represented by pneumatic commercial Flexcell^®^ device), particularly in generating biaxial (radial and circumferential) tensile strains. Depending on how the systems are configured, both positive and negative pressures can be employed. However, conventional configurations with hydrogels bounding on an elastomeric membrane could suffer from several limitations such as non-uniform strain distribution (strain magnitude changes with distance from the center of the membrane), limited oxygen and nutrient diffusion as hampered by the bounding membrane, and low throughput (Riehl et al., 2012). In addition, pneumatic actuator may be limited in actuation rate due to the effect of air compressibility.

With the development of microengineering technologies, significant progress has been made by combing elastomeric membranes and elastic micropillars. In this case, hydrogel microtissue constructs are not directly stick to the elastomeric membranes but constrained by the elastic micropillars. The distance between the micropillars changes when the underlying elastomeric membranes are stretched by either motor-driven (Asmani et al., 2018; Chen et al., 2019) or pneumatic actuation (Asmani et al., 2019; van Kelle et al., 2019), leading to deformation of the constrained microtissues. Alternatively, pneumatic actuation of micropillar-anchored microtissues can be also realized by applying vacuum pressure to the side chambers of microtissues (Walker et al., 2018, 2019), similar to the actuation system developed for 2D cell culture (Huh et al., 2010). These approaches allow simultaneous stretching of multiple microtissues, and enable the measurement of microtissue contraction forces at the same time by following micropillar deflection.

What is more, when fixing magnetic microspheres to the micropillars, the microtissues can be magnetically actuated in a non-contact and remote way by applying a non-uniform magnetic field to attract the microspheres (Zhao et al., 2013). This eliminates the actuation and even the use of elastomeric membranes. Similar approach has been developed by fabricating magnetic responsive layer onto photopatterned microtissues that are chemically anchored on glass substrates, which enables magnetic actuation without the complicated fabrication of micropillars (Li et al., 2016a,b). Custom magnetic tweezers have been used to selectively actuate a single microtissue or micropillar (Bose et al., 2018). To allow simultaneous actuation of multiple microtissues, custom-assembled magnet arrays and electrodeposited bar magnetics have been developed (Xu et al., 2015; Li et al., 2016b). Compared to motor-driven approach and pneumatic actuation, magnetic actuation brings some additional important benefits such as the ability to measure microtissue stiffness and to change boundary stiffness (i.e., the effective bending stiffness of the micropillars) in real time (Zhao et al., 2014; Kural and Billiar, 2016; Rodriguez et al., 2019). A potential limitation for magnetic actuation is that it could be cumbersome to design and integrate the magnetic actuation system. In addition, a strong magnetic field could be required in certain conditions to achieve high strain amplitude, which may impose unwanted magnetic effect on cells.

## Concluding Remarks and Perspectives

An increasing interest has been put on the engineering of 3D cell microenvironment. As an essential cue for the survival, growth and functional performance of many types of cells, mechanical stretch is required but difficult to control in three dimensions. While remarkable progresses have been made in recent years, much work remains to be done.

First, hydrogels used for supporting cell stretching and culture in 3D usually have too simplified composition and poor controlled properties. The influences of hydrogel composition and properties on cell responses to mechanical stretching have been seldom investigated, despite the fact that hydrogels can provide diverse important cues for regulating cell behaviors (Atance et al., 2004; Watson et al., 2014). Second, most of the current studies have used lab-specific stretching devices without strict control and characterization of strain profiles in hydrogels, making it difficult to precisely regulate cell behaviors and compare the results of different studies. It is therefore necessary to develop standardized stretching devices that can be easily adopted by different labs for programmable mechanical stretching of cells in three dimensions. Third, most of the reported stretching devices are designed to induce a single stretch parameter at a time, limiting simultaneous investigation of multiple stretch parameters. Although several stretching devices have been designed to simultaneously apply multiple stretch parameters, media is usually pooled among samples in these studies, complicating data analysis with paracrine signaling. Finally, methods based on advanced optical imaging have been widely used in characterizing cell responses to mechanical stretching (Fu et al., 2018). However, other methods such as those based on electrochemical (Liu Y. L. et al., 2016; Liu et al., 2017; Liu et al., 2019; Huang and Liu, 2020), impedance (Lind et al., 2017; Zhang et al., 2017; Liu et al., 2018), and current (Mannhardt et al., 2019) sensing, once extended, may be particularly useful in fast, real-time and high-content analysis of biological and mechanical events.

Currently, mechanical stretching has greatly promoted the engineering of 3D tissue constructs for regeneration, however, mechanotransduction studies on cell stretching have been mostly performed in two dimensions, thus limiting in-depth understanding of stretch mechanobiology in pathophysiological conditions. Moreover, mechanical stretching has been seldom considered in engineered microtissues that used for drug screening applications. Future efforts should be directed toward the development of more versatile and predictive 3D cell stretching models for mechanobiology and drug screening studies. We anticipate that it will be promising to integrate and take advantages of advanced biomaterials, actuators and microengineering technologies such as 3D printing, microfluidics, and microelectromechanical systems.

## Author Contributions

All authors listed have made a substantial, direct and intellectual contribution to the work, and approved it for publication.

## Conflict of Interest

The authors declare that the research was conducted in the absence of any commercial or financial relationships that could be construed as a potential conflict of interest.
